# Early functional network alterations in asymptomatic elders at risk for Alzheimer’s disease

**DOI:** 10.1038/s41598-017-06876-8

**Published:** 2017-07-26

**Authors:** Akinori Nakamura, Pablo Cuesta, Takashi Kato, Yutaka Arahata, Kaori Iwata, Misako Yamagishi, Izumi Kuratsubo, Kimiko Kato, Masahiko Bundo, Kersten Diers, Alberto Fernández, Fernando Maestú, Kengo Ito

**Affiliations:** 1 Department of Clinical and Experimental Neuroimaging, Center for Development of Advanced Medicine for Dementia, National Center for Geriatrics and Gerontology, Obu, Japan; 2 National Hospital for Geriatric Medicine, National Center for Geriatrics and Gerontology, Obu, Japan; 3 Laboratory of Cognitive and Computational Neuroscience, Center for Biomedical Technology, Complutense University of Madrid and Technical University of Madrid, Madrid, Spain; 4 Department of Basic Psychology II, Complutense University of Madrid, Madrid, Spain; 5 Department of Psychiatry, Faculty of Medicine, Complutense University of Madrid, Madrid, Spain; 6 Department of Psychology, Technische Universität Dresden, Dresden, Germany

## Abstract

Amyloid-β (Aβ) deposition is known to starts decades before the onset of clinical symptoms of Alzheimer’s disease (AD), however, the detailed pathophysiological processes underlying this preclinical period are not well understood. This study aimed to investigate functional network alterations in cognitively intact elderly individuals at risk for AD, and assessed the association between these network alterations and changes in Aβ deposition, glucose metabolism, and brain structure. Forty-five cognitively normal elderly subjects, who were classified into Aβ-positive (CN+) and Aβ-negative (CN−) groups using ^11^C-Pittsburgh compound B PET, underwent resting state magnetoencephalography measurements, ^18^F-fluorodeoxyglucose PET (FDG-PET) and structural MRI. Results demonstrated that in the CN+ group, functional connectivity (FC) within the precuneus was significantly decreased, whereas it was significantly enhanced between the precuneus and the bilateral inferior parietal lobules in the low-frequency bands (theta and delta). These changes were suggested to be associated with local cerebral Aβ deposition. Most of Aβ+ individuals in this study did not show any metabolic or anatomical changes, and there were no significant correlations between FC values and FDG-PET or MRI volumetry data. These results demonstrate that functional network alterations, which occur in association with Aβ deposition, are detectable using magnetoencephalography before metabolic and anatomical changes are seen.

## Introduction

Alzheimer’s disease (AD) is the most common cause of dementia, which is considered as a continuum of preclinical, prodromal and dementia stages^1, 2^. Along with the recent disease-modifying clinical trials that showed potential efficacy^3, 4^, the importance of earlier intervention, preferably before the progression of significant neuronal death, has been intensified. Therefore, studies that investigate pathophysiological changes in the preclinical stages of AD are becoming more important. β-amyloid (Aβ) plaques and neurofibrillary tangles are pathognomonic signatures of AD, and the accumulation of Aβ is known to start decades before the onset of the dementia stage^5–7^. Several studies have demonstrated that about 20–40% of cognitively intact individuals aged 60–90 years have Aβ deposition^8^. In these individuals, termed as “preclinical AD”^9^ or “asymptomatic at risk for AD”^2^, “downstream” markers that can serve as surrogate markers for disease progression have been extensively studied. These investigations have demonstrated that individuals with positive Aβ signatures, in longitudinal studies, showed increased concentrations of CSF total tau and phosphorylated tau^1^, reduced fluorodeoxyglucose (FDG) uptake predominantly in the posterior cingulate, precuneus, and temporo-parietal cortices^10^, a faster rate of brain atrophy, especially in the medial temporal area^11, 12^, abnormal default mode network (DMN)^13^ function as measured by fMRI^14–20^, and accelerated cognitive decline^21, 22^. According to the hypothetical model of the pathological cascade of AD^23^, the markers downstream to the Aβ deposition are CSF tau, metabolic and structural changes as shown on FDG-PET and structural MRI (sMRI), and then cognitive impairments in temporal order. The National Institute on Aging and the Alzheimer’s Association (NIA-AA) (2011) have proposed a conceptual framework for preclinical AD consisting of 3 stages: Stage 1 for asymptomatic amyloidosis, Stage 2 for amyloidosis plus evidence of neurodegeneration including neuronal dysfunction detected by FDG-PET or fMRI, or cortical thinning or hippocampal atrophy detected by sMRI, and Stage 3 for Stage 2 plus subtle cognitive decline^9^. However, these models are yet not settled^2^, and detailed pathophysiological processes during the long-lasting preclinical period are still not fully understood. We expect that magnetoencephalography (MEG) will provide new insights to identify unique topographical downstream markers to monitor synaptic functional alterations for the following reasons: First, MEG measures the postsynaptic potential, which is the primary signal of neuronal activity^24^. Second, with its temporal resolution of milliseconds, the neuronal oscillatory activity can be analyzed in detail^25, 26^. Third, with its decent spatial resolution, MEG can provide topographical information. Therefore, the main objective of this study was to use MEG to investigate functional network alterations associated with the cerebral Aβ deposition in asymptomatic individuals at risk for AD. We analyzed functional connectivity (FC) of the resting state MEG signals in 32 amyloid-negative and 13 amyloid-positive cognitively intact elders who were classified based on Pittsburgh Compound B (PiB) PET imaging. We also analyzed the correlations between FC changes and amyloid deposition. Further, we analyzed other downstream markers provided by FDG-PET and sMRI data, and compared findings with the results of MEG. Several MEG/EEG studies report neural network disruption in AD and mild cognitive impairment (MCI)^27–31^; however, no report assesses the relationship between electrophysiological FC and amyloid-PET, FDG-PET, and sMRI in the preclinical stage of AD.

## Materials and Methods

### Participants

This study was a part of the MULNIAD study (Multimodal NeuroImaging for Alzheimer’s disease Diagnosis), which is a prospective longitudinal study targeting normal aging, MCI, and Alzheimer’s disease, conducted at the National Center for Geriatrics and Gerontology (NCGG). The study was approved by the Ethics Committee of NCGG, and all participants provided written informed consent. All methods were performed in accordance with the Declaration of Helsinki and “Ethical Guidelines for Medical and Health Research Involving Human Subjects” issued by the Ministry of Health, Labour and Welfare in Japan. First, we identified 68 cognitively normal (CN) individuals and then selected 13 PiB-positive (CN+) individuals based on visual ratings of PiB-PET images, as described below. Then we selected 32 PiB-negative (CN−) individuals so that age, sex, and education were matched. Thus, we analyzed 45 participants aged 64 to 79 years (mean, 75.2 ± 4.7 years; 20 males). All subjects were native Japanese participants recruited mainly from community-dwelling people (Table 1). All of them were right handed except for one in the CN− group who was classified as mixed-handed according to the Edinburgh handedness inventory^32^. To define the CN individuals, we followed the inclusion criteria of the Alzheimer’s Disease Neuroimaging Initiative 2 (ADNI2) study (http://adni.loni.usc.edu/) (Supplementary Methods).

**Table 1:** Demographics of the participants.

	CN+ (n = 13)	CN− (n = 32)	Statistics*p*-value	Effect size *φ*/Cohen’s *d*
Sex (M: F)	7:6	13:19	0.42	0.12
Age (y)	71.85 ± 4.39	70.97 ± 4.25	0.54	0.21
Education (y)	12.38 ± 3.15	11.87 ± 2.63	0.58	0.18
MMSE	28.77 ± 1.09	28.88 ± 1.34	0.80	0.09
ADAS-Jcog	5.74 ± 2.19	5.66 ± 2.6	0.92	0.03
LM1	20.77 ± 7.25	20.84 ± 6.04	0.97	0.01
LM2	16.23 ± 6.85	16.97 ± 6.21	0.73	0.12
CDR	0	0	—	—
CDR SOB	0.04 ± 0.14	0.08 ± 0.18	0.49	0.24
GDS	2 ± 1.29	2.13 ± 1.68	0.81	0.08
APOEε4 (%)	4/13 (30.8%)	6/32 (18.8%)	0.38	0.13
PiB-mcSUVR *	1.41 ± 0.18	1.12 ± 0.05	<0.001	2.75
PiB-DMNSUVR *	1.52 ± 0.22	1.13 ± 0.06	<0.001	3.07
FDG-PET score	0.47 ± 0.18	0.50 ± 0.28	0.71	0.12
VSRAD *z*-score	0.54 ± 0.18	0.67 ± 0.41	0.31	0.34

Values are presented as mean ± SD. Statistical analyses were performed using the chi square test (sex, APOE) and Student’s *t*-test (others). The asterisks indicate statistically significant group differences. The effect sizes were computed as *φ* (sex, ApoE) or Cohen’s *d* (others).

MMSE: Mini-Mental State Examination, ADAS-Jcog: Alzheimer’s Disease Assessment Scale-Cognitive Component-Japanese version, LM1/LM2: Logical Memory I/II from the Wechsler Memory Scale–Revised (paragraph A and B), CDR: Clinical Dementia Rating, SOB: Sum of Boxes, GDS: Geriatric Depression Scale, APOEε4: positive for apolipoprotein Eε4, PiB-mcSUVR: mean cortical standardized uptake value ratio (SUVR) of PiB-PET, PiB-DMNSUVR: mean SUVR values within the 6 default mode network (DMN)-related regions of interest, FDG-PET score: a score that indicates the severity of the metabolic decrease in brain areas typically affected by Alzheimer’s disease, VSRAD z-score: the degree of gray matter atrophy of the medial temporal region using a z-score computed by Voxel-based Specific Regional Analysis System for Alzheimer’s Disease.

All participants underwent comprehensive neuropsychological batteries, and neuroimaging assessments including PiB-PET, FDG-PET, structural MRI, and MEG. All examinations were carried out within about 1 month of each other.

### PiB-PET

#### Image acquisition

3D PET imaging for 50–70 min after intravenous injection of 555 ± 185MBq ^11^C-PiB was carried out using a PET CT camera, Biograph True V (Siemens Healthcare, Erlangen, Germany). X-ray CT for attenuation correction was performed before PET imaging.

#### Visual rating and classification

The visual rating of PiB-PET images was conducted as previously reported^33^ and used for classification of participants into PiB-positive (CN+) and PiB-negative (CN−) groups (Supplementary Methods).

#### Quantitative image analysis

The standardized uptake value ratio (SUVR) images were generated individually using the Harvard-Oxford probabilistic atlas^34^. Mean cortical SUVR (PiB-mcSUVR) values were obtained by averaging the SUVRs of the frontal, parietal, and temporal areas (Supplementary Methods). In addition, mean SUVR values within each of the 6 DMN-related region of interest (ROI) (see below *MEG data analysis*) (PiB-LocalSUVR), and mean values of these 6 ROIs (PiB-DMNSUVR) were also obtained. Voxelwise regression analysis for MEG FC data with the PiB-SUVR images was performed using Statistical Parametric Mapping (SPM8, Wellcome Trust Centre for Neuroimaging, University College, London, UK) (Supplementary Methods).

### FDG-PET

#### Image acquisition

Using the same scanner and the attenuation correction method as PiB-PET, ^18^F-FDG-PET images were obtained (Supplementary Methods).

#### Image interpretation

The FDG-PET images were processed with the 3-dimensional stereotactic surface projections (3D-SSP) technique to generate z-score maps, using iSSP software version 5 (Nihon Medi-Physics Co. Ltd., Tokyo, Japan). The normal database used for generating the z-score maps was constructed at NCGG using 40 normal elderly subjects (a 20 age-matched subjects’ dataset was used for each analysis). The two experts who provided the PiB visual rating visually interpreted the 3D-SSP images to determine whether a subject showed a specific pattern of decreased glucose metabolism in the precuneus/posterior cingulate and temporo-parietal areas (Supplementary Methods).

#### Quantitative analysis using FDG-PET score

For quantitative assessments of FDG-PET images, we computed PET scores as previously reported^35^. At first, we calculated the sum of *t*-values in predefined areas that are typically affected by Alzheimer’s disease (AD *t*-sum scores)^36^, which indicate the severity of the metabolic decrease in brain areas typically affected by Alzheimer’s disease. Then the AD *t*-sum score was converted into the PET score by reference to its upper limit of normal and log transformation to approach a normal distribution of values, according to the following equation: PET score = log2 {(AD *t*-sum/11,089) + 1}^37^.

### Structural MRI

#### Image acquisition

High-resolution 3D T1-weighted images were acquired using a Trio 3 T scanner (Siemens), and used for volumetric analysis (Supplementary Methods). T2-weighted and fluid attenuated inversion recovery images were also acquired to assess brain lesions.

#### Volumetric analysis

Atrophy in the medial temporal region, including the hippocampus, was assessed using the Voxel-based Specific Regional Analysis System for Alzheimer’s Disease (VSRAD^®^ advance, Eisai Co., Ltd., Tokyo, Japan) software, which is based on SPM8 plus DARTEL^38, 39^. The VSRAD outputs the degree of gray matter atrophy of the medial temporal region using a *z*-score by comparing it with a normal database comprised of 60 healthy volunteers (27 males and 33 females) aged 60 to 89 years (mean, 74.1 ± 8.2 years) independently obtained at NCGG. The spatially normalized and gray matter-segmented images created by VSRAD were also used for the whole-brain voxel-based morphometry of group comparisons using SPM8. In addition, ROI-based volumetric analyses were also carried out using the same spatially normalized and gray matter-segmented images and MarsBaR toolbox (http://marsbar.sourceforge.net/). The mean values of normalized gray matter volume within the 6 DMN-related ROIs were extracted and used for the ROI-based analyses.

### MEG

#### Measurements

MEG measurements were performed using a 306-channel whole-head MEG system (Vectorview, ElektaNeuromag, Finland), which was placed in a magnetically shielded room at NCGG. Participants sat comfortably on a chair with their eyes closed, and 5 minutes of resting state MEG signals were measured with a sampling rate of 1000 Hz (online bandpass anti-alias filtering at 0.1–330 Hz) (Supplementary Methods). The arousal level of each subject was monitored through a video camera (WV-CL934, Panasonic, Japan), and also checked via a conversation immediately after the measurement session. If a subject reported he/she felt sleepy during the session, we gave him/her sufficient time to become more awake and redid the measurement.

#### Data analysis

After the data preprocessing (Supplementary Methods), at least 20 artifact-free fragments (trials) of continuous 4-second MEG signals (80 seconds of brain activity) were obtained from all participants. Then the trials were filtered in the following frequency bands: delta (2–3.9 Hz), theta (4.1–7.9 Hz), alpha (8.1–11.9 Hz), beta (12.1–29.9 Hz), and gamma (30.1–55.0 Hz).

Using the realistic single-shell model with a 1-cm spacing grid (2455 nodes), the source reconstruction was performed using a Linearly Constrained Minimum Variance Beamformer^40^ (Supplementary Methods).

**Figure 1:**
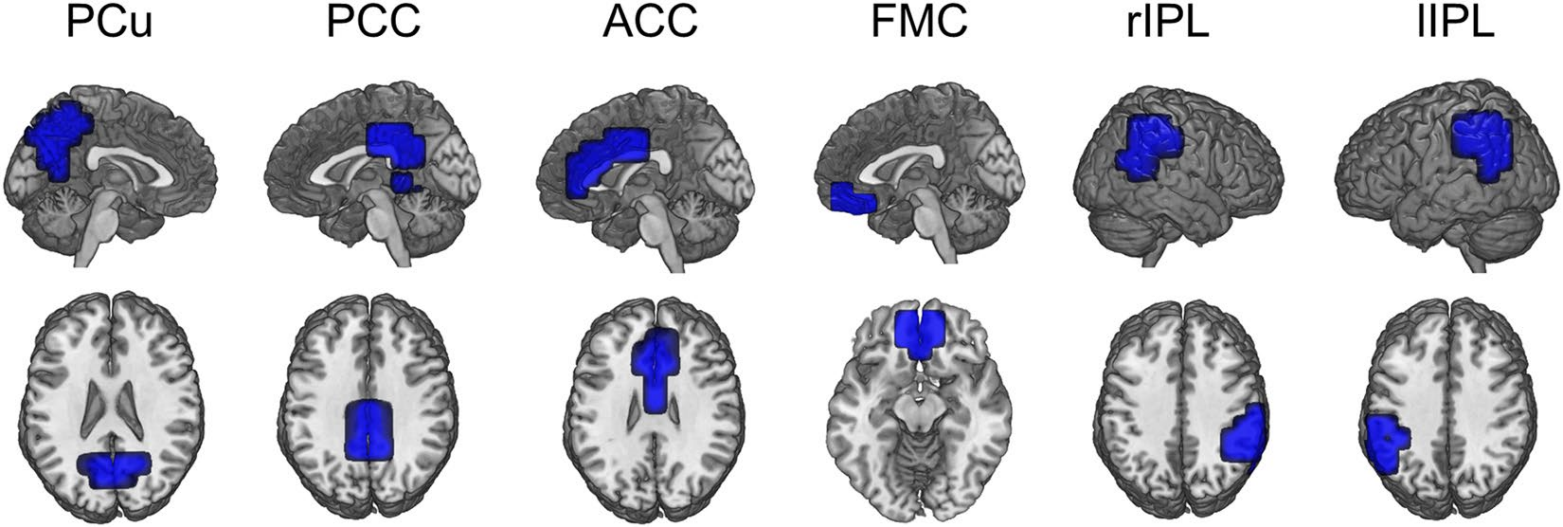
Shapes for the six ROIs related to the default mode network (DMN). The medial parts of the brain (each of the bilateral PCu, PCC, ACC and FMC) were merged into a single ROI. In addition, the right or left supramarginal and angular cortices were merged as rIPL or lIPL. PCu: precuneus, PCC: posterior cingulate cortex, ACC: anterior cingulate cortex, FMC: frontal medial cortex, rIPL: right inferior parietal lobule, lIPL: left inferior parietal lobule.

The FC analysis was performed using atlas-based ROIs. In this study, we specifically focused on FC in the DMN. We set six DNM-related ROIs in the precuneus (PCu), posterior cingulate cortex (PCC), anterior cingulate cortex (ACC), frontal medial cortex (FMC), and bilateral inferior parietal lobules (rIPL/lIPL) by referring to the Harvard-Oxford probabilistic atlas^34^ (Fig. 1). Then, the nodes that belong to each ROI (with at least a 25% probability) were selected. In total, 156 nodes were included in this study, as they were located within the six ROIs.

The FC was measured by means of phase-locking value (PLV), in each frequency band^41^ (Supplementary Methods).

Clusters of connections (network motifs) that showed statistically significant group differences (CN+ subjects vs CN− subjects) were explored by relying on the cluster-based permutation test^42^ for each frequency band. We analyzed both the intra-ROI FC, which computed the local connectivity within each ROI, and the inter-ROI FC, which evaluated the inter-regional connectivity from the PCu ROI to each other ROI. Only motifs that remained significant (*p* < 0.05) after correcting for multiple comparisons were further analyzed (Supplementary Methods). For the descriptive values of the significant motifs, we computed between-group statistics and effect sizes using the *t*-test and Cohen’s *d*.

Correlations between FC values and the PiB-SUVR values, FDG-PET scores, or VSRAD z-scores were assessed using the Pearson product-moment correlation coefficient analysis. Statistical analyses were done using Matlab R2009b (The Mathworks Inc, Natick, MA, USA) m and SPSS ver. 21 (IBM, Armonk, NY, USA). All tests were two-tailed, and the significance level was set at *p* < 0.05.

#### Data Availability

The datasets analyzed during the current study are available from the corresponding author on reasonable request.

## Results

### MEG FC Analysis

**Figure 2:**
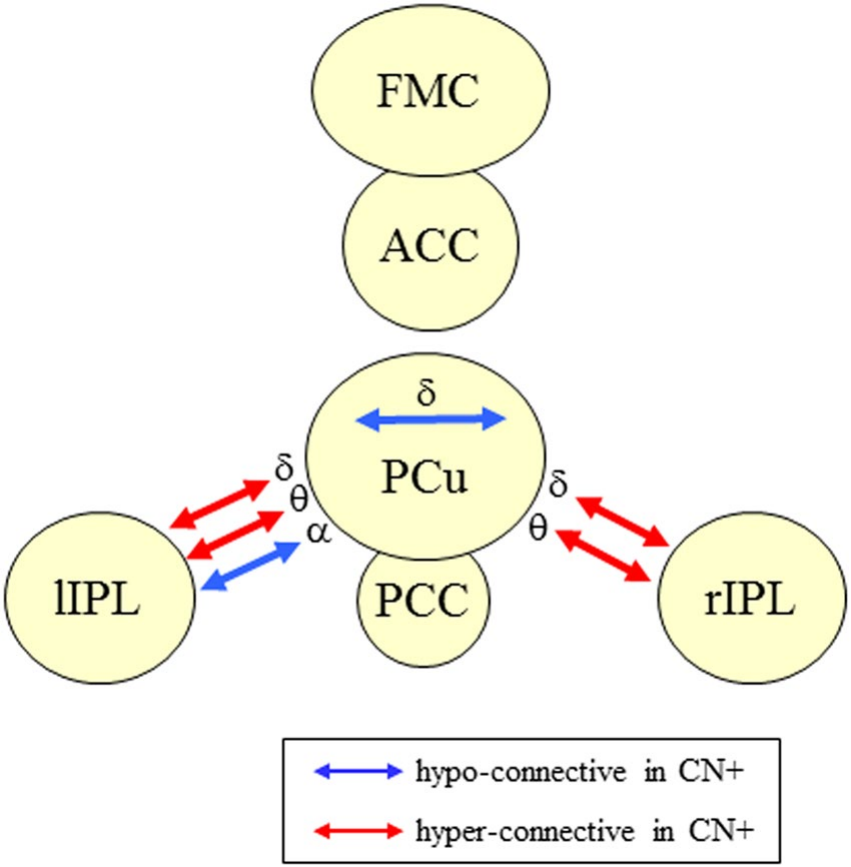
Summary of the network alteration in CN+ subjects. The network motifs that showed statistically significant differences in CN+ subjects compared with CN− subjects are shown. The blue and red arrows indicate hypo-connective motifs (CN+ subjects < CN− subjects) and hyper-connective (CN+ subjects > CN− subjects) motifs, respectively. The Greek characters indicate the frequency bands that showed significant differences (δ: delta, θ theta, α: alpha). CN+: cognitively normal PiB-positive, CN−: cognitively normal PiB-negative.

Results of the FC analysis are summarized in Table 2 and Fig. 2. The intra ROI FC analysis demonstrated that the local network connectivity, as evaluated by PLV, was significantly more diminished in CN+ subjects than in CN− subjects within the PCu ROI. This alteration was found in the delta band. We did not find any significant local FC changes in other ROIs.

**Table 2:** Results of the FC analysis in 13 CN+ and 32 CN− subjects.

	ROI	Bands	Number of links	PLV (mean ± SD)		*t*-test	Effect size	FC changes	Correlation with
			(motif size)	CN+	CN−	*p value* †	Cohen’s *d*	in CN+‡	DMNSUVR §
Intra ROI FC	PCu	Delta	39	0.433 ± 0.024	0.470 ± 0.022	<0.001	1.64	Hypo	−0.582***
Inter ROI FC	PCu - rIPL	Delta	47	0.366 ± 0.008	0.343 ± 0.011	<0.001	2.24	Hyper	0.677***
		Theta	41	0.288 ± 0.015	0.265 ± 0.009	<0.001	2.16	Hyper	0.673***
	PCu - lIPL	Delta	29	0.365 ± 0.011	0.343 ± 0.010	<0.001	2.17	Hyper	0.614***
		Theta	37	0.287 ± 0.009	0.265 ± 0.010	<0.001	2.31	Hyper	0.704***
		Alpha	39	0.264 ± 0.012	0.286 ± 0.014	<0.001	1.72	Hypo	−0.473**

^†^The *p* values were Bonferroni corrected by multiplying by 5 (the number of frequency bands). ^‡^FC changes in the CN+ group compared with the CN− group. Hypo, decreased connectivity in the CN+; Hyper, increased connectivity in the CN+. §Correlation coefficient (*r*) for each FC value with PiB mean standardized uptake value ratio (SUVR) value within the default mode network (DMN) ROIs (DMNSUVR). The asterisks indicate statistically significant correlations (*p < 0.05, **p < 0.01, ***p < 0.001). PCu: precuneus, rIPL: right inferior parietal lobule, lIPL: left inferior parietal lobule.

In the inter ROI FC analysis, we found that the long-distance network connectivity was also altered in CN+ subjects. Significant FC differences between the two groups were found in the PCu-rIPL and PCu-lIPL connections in several frequency bands. Most of these alterations were enhanced (hyper) connectivity in CN+ subjects compared with CN− subjects, except the alpha band in the PCu-lIPL connection, which showed diminished (hypo) connectivity in CN+ subjects.

### Correlation between MEG FC data and PiB-PET

**Figure 3:**
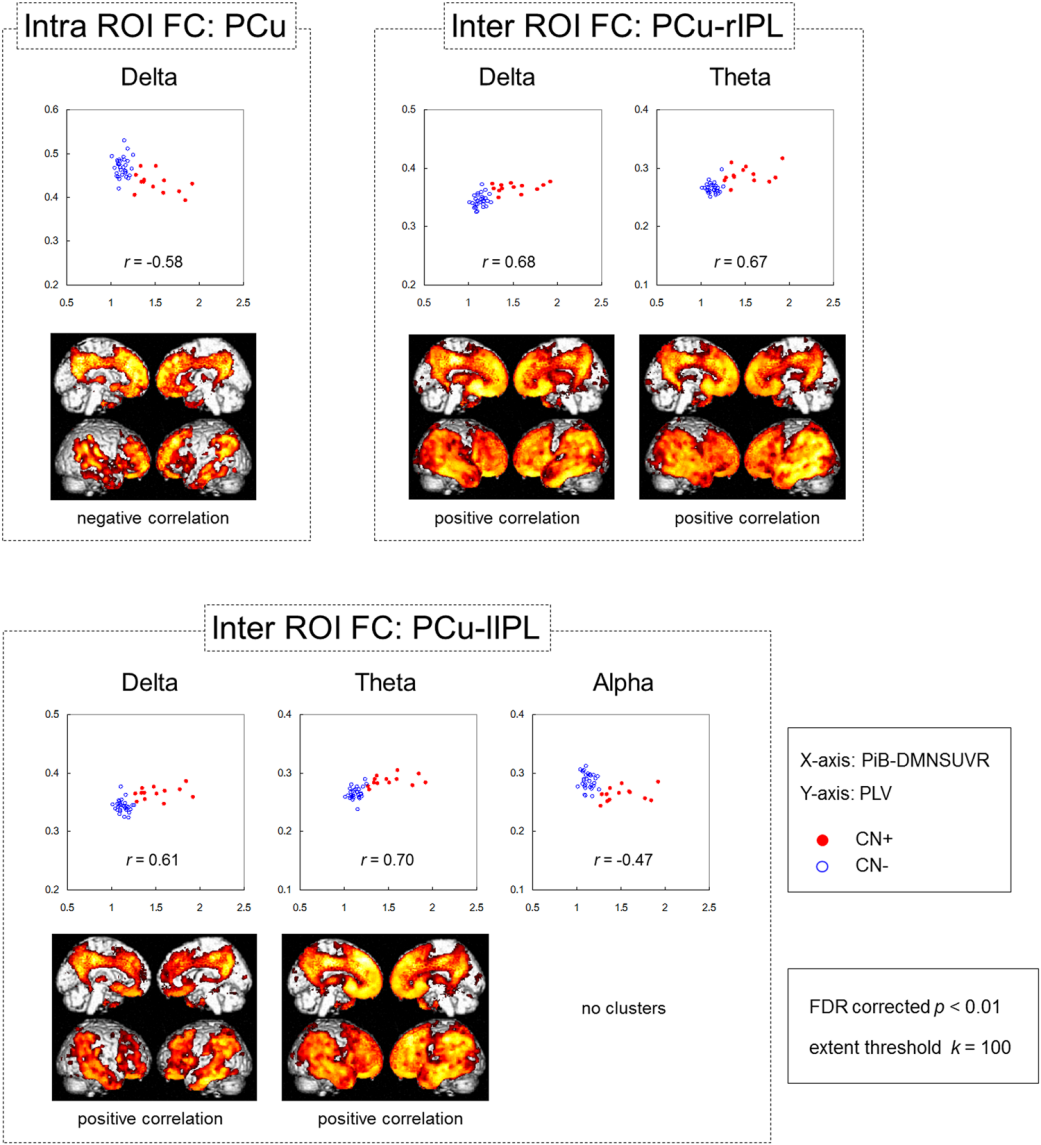
Correlations between each MEG FC marker and cerebral amyloid deposition. The scatter plots show correlation between the PiB-DMNSUVR values (x axis) and PLV (y axis). The closed red circles and open blue circles indicate CN+ and CN− individuals, respectively. The brain images display the results of regression analysis between PiB-SUVR images and each MEG FC marker’s PLV. Brain areas that showed statistically significant correlations between regional PiB retention and each biomarker are visualized. The height threshold is *p* < 0.01 (FDR corrected), and the extent threshold is *k* = 200 voxels in all images. Note that the regression analyses for PCu-lIPL alpha showed no significant clusters with the above thresholds. FC: functional connectivity, DMNSUVR: mean standardized uptake value ratio (SUVR) within the default mode network (DMN). PLV: phase-locking value, CN+: cognitively normal PiB-positive, CN−: cognitively normal PiB-negative, FDR: false discovery rate, PCu: precuneus, lIPL; left inferior parietal lobule.

To assess the relationships between the network alteration and the cerebral amyloid deposition, we analyzed correlations between FC values (henceforth MEG FC markers) and PiB-DMNSUVR values. At first, we performed multiple regression analyses to evaluate the influence of possible confounders that may affect the correlation between each MEG FC marker and PiB-DMNSUVR values. The results demonstrated that neither age, years of education, sex, nor APOE ε4 showed significant effects. Therefore, we conducted simple correlation analyses. All MEG FC markers showed significant negative or significant positive correlations with PiB-DMNSUVR values (Table 2). Figure 3 shows the scatter plots of the MEG FC markers and PiB-DMNSUVR values, and results of regression analyses between the PiB-SUVR images and each MEG FC marker. These results demonstrated that FC markers, especially in the low-frequency bands (delta and theta), appeared to be linearly correlated with cerebral amyloid deposition.

**Figure 4:**
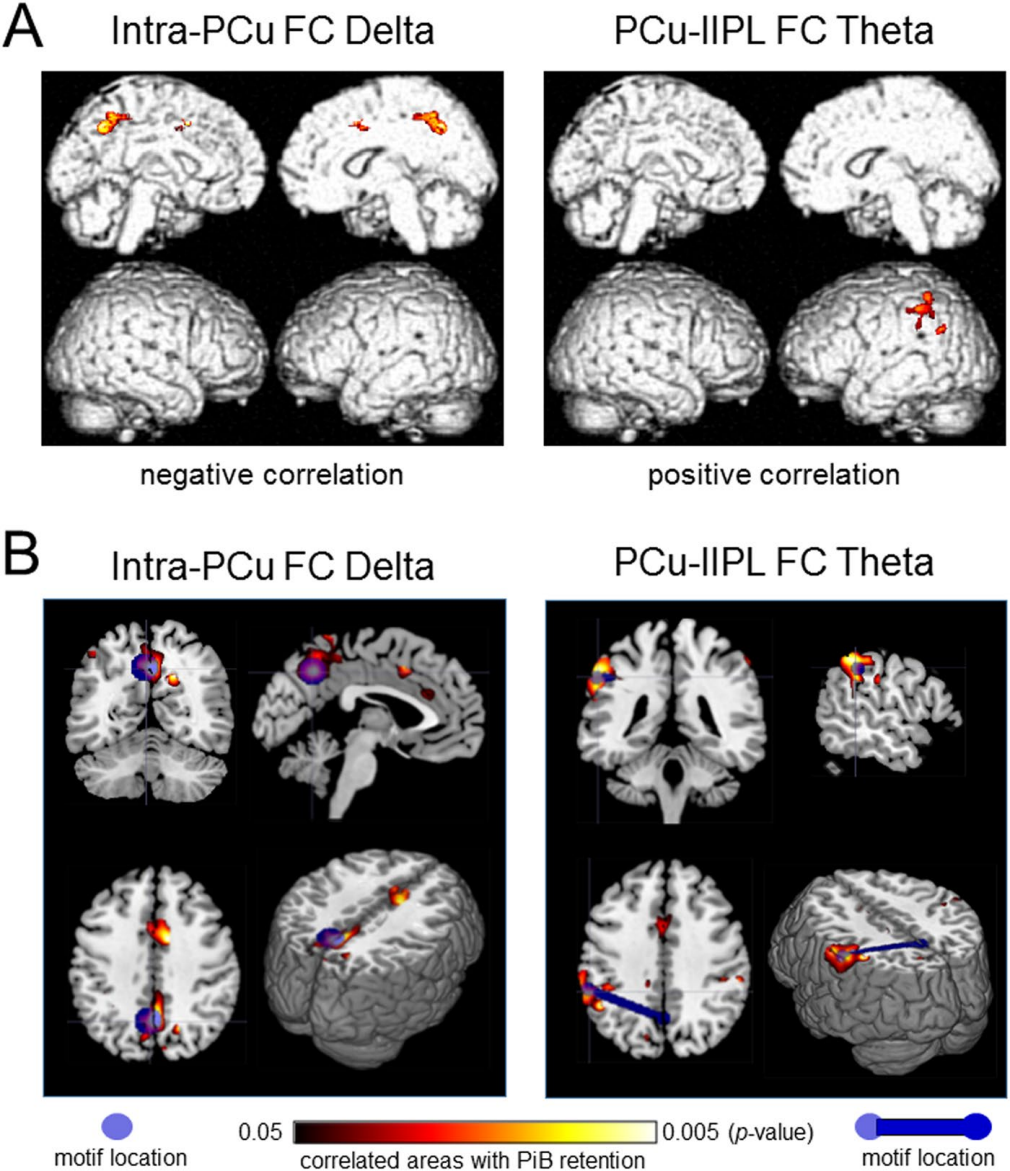
Results of the within-group correlation and regression analyses. (**A**) Results of regression analysis between PiB-SUVR images and values of MEG FC markers. Correlated areas within a DMN-ROI mask are visualized. The height threshold is *p* < 0.01 (uncorrected), and the extent threshold is *k* = 100 voxels in all images. (**B**) Topographical relationships between the network motif and the correlated areas between the FC values and PiB retention. The motif locations are represented by the center of gravity at each ROI. SUVR: standardized uptake value ratio, FC: functional connectivity, DMN: default mode network.

To further ensure the relationship between the MEG FC markers in the low frequency bands and local amyloid deposition, we conducted correlation analyses separately within each group (CN+ and CN−). The results showed that 1) The within-group correlations were generally higher in CN+ than those in CN−, and 2) if we restricted the SUVR values to more locally associated ROIs (PiB-LocalSUVR), the correlations became higher in CN+ (*p* < 0.05, permutation test) (Table 3). For the LocalSUVR values, all five MEG FC markers showed a correlation coefficient greater than 0.3 (in absolute value) in CN+, suggesting some dose-dependent effects. In particular, PCu-lIPL FC in the theta band showed a statistically significant correlation (*r* = 0.55, *p* = 0.049), and the intra-PCu FC in the delta band also showed a similarly high correlation coefficient (*r* = −0.53, *p* = 0.063). For these two MEG FC markers, we further conducted regression analyses to PiB-PET images using SPM8. The results are shown in Fig. 4A. For the intra-PCu delta FC, the correlated areas were mainly clustered in the PCu, and for the PCu-lIPL theta FC, they were clustered in the left IPL. Moreover, if the network motif of each MEG marker’s connection was overlaid on the results of the regression analysis, the location of the motif and the cluster of the correlated area were overlapped well (Fig. 4B), indicating close topographical association between local amyloid deposition and local FC changes. Because the within-group correlation analysis sacrifices both the sample size and the width of the range, dismissing the control values, only one FC motif reached significance in the simple correlation analysis (Table 3). However, together with all of the above results, it would be reasonable to consider that the FC changes in the low-frequency bands, at least the Intra-PCu FC in the delta and PCu-lIPL in the theta, were associated with local amyloid deposition.

**Table 3:** Correlation between PiB-SUVR values and FC within each group.

	ROI	Bands	DMNSUVR (*r*)		LocalSUVR (*r*) †	
			CN+	CN−	CN+	CN−
Intra ROI FC	PCu	Delta	−0.432	0.091	−0.529	0.127
Inter ROI FC	PCu - rIPL	Delta	0.287	0.307	0.352	0.290
		Theta	0.291	0.132	0.297	0.266
	PCu - lIPL	Delta	0.279	−0.191	0.395	−0.200
		Theta	0.335	0.333	0.554*	0.289

*p < 0.05. ^†^LocalSUVR: standardized uptake value ratio (SUVR) values for more local ROIs were used for the correlation analysis. For intra ROI FC at PCu, SUVR values in the PCu ROI were used. For inter-ROI FC, SUVR values in the rIPLand lIPL were used for the PCu-rIPL and PCu-lIPL connections, respectively.

### Other imaging markers (FDG-PET and structural MRI)

**Figure 5:**
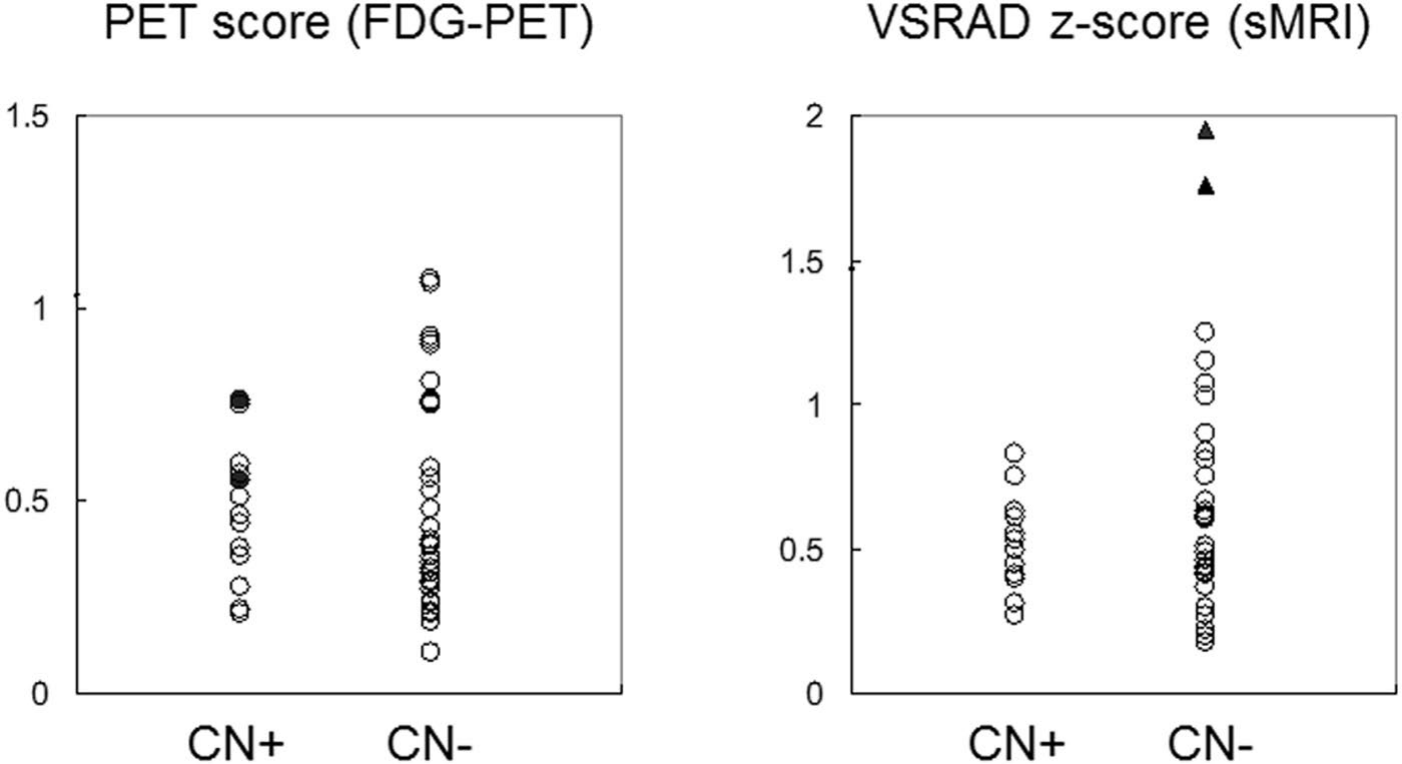
Scatter plots of the quantitative image analyses. Left: FDG-PET scores in CN+ subjects and CN− subjects. The closed circles indicate two cases who were visually rated as having probable Alzheimer’s disease-like metabolic changes. Right: VSRAD z-scores in the two groups. The closed triangles indicate the two outliers defined by the Tukey method. CN+: cognitively normal PiB-positive, CN−: cognitively normal PiB-negative, VSRAD: Voxel-based Specific Regional Analysis System for Alzheimer’s Disease.

The visual interpretation of the FDG-PET images detected that two of 13 subjects in the CN+ group had probable decreased glucose metabolism in the PCu/PCC and temporo-parietal areas. However, the quantitative analysis using FDG-PET score showed there were no significant group differences between CN+ and CN− subjects (Table 1 FDG-PET score, and Fig. 5, left). There was no significant correlation between the FDG-PET score and PiB-mcSUVR or PiB-DMNSUVR values. Also, none of the MEG FC markers was significantly correlated with the FDG-PET score. The group comparison of the whole-brain FDG-PET images using SPM8 did not show any differences in regional glucose metabolism (FDR corrected p = 0.05, extent threshold k = 100).

The cortical volume in the medial temporal region was assessed using VSRAD. There were no group differences in the z-score (Table 1 and Fig. 5, right). None of the participants showed a z-score more than 2.0, which is the cut-off line for significant atrophy in the medial temporal region (Matsuda *et al*., 2012; Matsuda, 2013). In particular, z-scores of all CN+ subjects were less than 1.0, indicating that there was no CN+ individual who showed quantitatively detectable cortical volume loss in the medial temporal region. Conversely, there were two outlier subjects (Tukey method for outliers) in the CN− group showing a z-score more than 1.5 (Fig. 5, right). The VSRAD z-score was not significantly correlated with either the PiB-mcSUVR value or the PiB-DMNSUVR value. None of the MEG FC markers was significantly correlated with the VSRAD z-score. The group comparison of the whole-brain voxel-based morphometry using SPM8 did not show any differences in the gray matter and white matter volume (FDR corrected p = 0.05, extent threshold k = 100). The ROI-based volumetric analyses also did not show any group differences in the gray matter volume (Supplementary Fig. S1 and Supplementary Table S1). Further, additional analyses using the cortical volume within the DMN-related ROIs as confounding covariates demonstrated quite similar results with those presented in Table 2 (Supplementary Table S2). These results indicated that the local cortical volume had no significant effects on the MEG FC markers.

## Discussion

This study demonstrates that the electrophysiological network activity in the DMN was altered in cognitively normal individuals at risk for Alzheimer’s disease, and these changes were associated with cerebral amyloid deposition. These results suggest that the electrophysiological FC, as measured by MEG, could potentially be a sensitive biomarker for the preclinical stage of Alzheimer’s disease. This is in line with the International Working Group’s expectation; “the pathologic changes that occur in the brain many years before the clinical onset should induce functional changes in brain structures, which can be identified by EEG/MEG”^43^.

The intra-ROI FC analysis showed that the local connectivity within the PCu, which is one of the typical regions for the Aβ accumulation^44^ and also known as the posterior hub of the DMN^13^, was significantly decreased in CN+ individuals in the delta band. In addition, the FC values were negatively correlated with the local cerebral amyloid deposition as represented by PiB-PET. These results agree with previous studies using resting state FC measured with fMRI in healthy elders^14–16, 18^, and are also consistent with animal studies that showed that Aβ accumulation disrupted the functional connectivity at the local level^45^. Although the relationship between Aβ deposition and synaptic dysfunction remains uncertain, the strong influence of Aβ in the destabilization of the cortical network activity has been noted by Palop and Mucke (2010)^46^. They indicated that molecular, cellular, and functional mechanisms within various forms of Aβ can lead to neuronal dysfunction (synaptic transmission, synaptic plasticity) in Alzheimer’s disease. These mechanisms may affect the local neuronal network organization, and consequently the network functional integrity, in individuals at risk for Alzheimer’s disease.

In contrast, the inter-ROI FC analysis showed that the long-distance connectivity in the low-frequency bands within the DMN was generally enhanced in CN+ individuals. Such a hyper-synchronous profile was also reported in several previous studies^16, 19, 47^, and has usually been explained as a compensatory mechanism. In our cases, the disrupted local network may cause up-regulation of the long-distance networks to maintain brain function. The neuronal hyper-synchronization can be also explained as an early aberrant excitatory response by the direct effect of local Aβ accumulation. Bousche *et al*. (2008)^48^ demonstrated that clusters of neurons near amyloid plaques become hyperactive. They suggested that the hyperactivity was caused by a relative decrease in synaptic inhibition. This finding was reinforced by a histological study by García-Marín *et al*. (2009)^49^ showing a reduction of inhibitory neurons in the vicinity of amyloid plaques. It is interesting that both hyper- and hypo-synchronization coexisted within the DMN in our study. However, it has been often reported that these two profiles were found among different DMN regions^14, 15, 19, 20, 50^, and among different brain networks^19, 20^ in cognitively intact elders.

There were considerable hemispheric differences in MEG FC markers. In the right hemisphere, FC was enhanced in the low-frequency bands. In the left hemisphere, it was also enhanced in the low-frequency bands, but diminished FC was found in the alpha band. This hemispheric asymmetry cannot be explained by a handedness factor because there was only one mixed-handed subject while all other subjects were right-handed, and subanalyses by eliminating the mixed-handed subject did not change the results. We speculate this finding might be due to the asymmetrical amyloid deposition in our subjects. The group comparison of the PiB-SUVR image showed that amyloid deposition is more robust in the left than the right hemisphere, including the left IPL (Supplementary Fig. S2). Due to the greater amyloid load, the PCu-lIPL connection might be affected specifically in the alpha band, which is the basic EEG rhythm. Then, as a compensatory mechanism, connections with other frequency bands in the left hemisphere were upregulated. Also, in the less-affected right hemisphere, upregulation might also occur to compensate for the more-affected left hemisphere. However, because the correlation between the PCu-lIPL FC in the alpha band and local amyloid deposition was not apparent, there may be alternative explanations.

The FC analysis using MEG allows the assessment of network functional integrity in multiple frequency domains. In this study, the enhancement of the inter-regional FC (PCu-rIPL and PCu-lIPL) in the delta and theta bands appeared to be the best MEG FC markers. They generally showed a larger effect size to discriminate the CN+ and C− groups. In particular, the PCu-lIPL in the theta band showed significant correlation with local amyloid deposition. Yet we do not know why the enhanced FC was dominant in the low-frequency bands. One possible explanation might be as follows: it is well-known that patients with Alzheimer’s disease and MCI generally show a “slowing” pattern consisting of increased EEG-MEG spectral power and FC in the slow frequency bands^26, 27, 51^. Our results might represent the very early stage of these EEG changes in Alzheimer’s disease.

One of the most important findings in this study is that the electrophysiological FC changes were clearly detected in CN+ individuals, despite the fact that most of them did not show any metabolic or anatomical changes. In addition, FC changes were correlated with amyloid deposition but not correlated with metabolic or anatomical changes. This is not consistent with the previous resting state fMRI study reported by Drzezga *et al*. that FC changes were positively correlated with regional glucose metabolism^18^. We consider this may be due to the different subject population used in our study. Their study included PiB-positive MCI subjects in addition to PiB-negative and -positive healthy controls, and regional glucose metabolism was significantly decreased in both PiB-positive healthy controls and MCI groups compared with the PiB-negative healthy controls^18^. In addition, a greater population of CN+ subjects in our study appears in the earlier preclinical stage than in their study because there were no differences in FDG-PET score between the CN+ and CN− groups. Even by careful visual interpretation, which is considered to be more sensitive than the FDG-PET score^35^, only two of 13 CN+ subjects showed a probable Alzheimer’s disease-like decrement pattern in regional glucose metabolism. Further, none of the CN+ subjects showed any detectable medial temporal atrophy. If we eliminated the two CN+ individuals with probable FDG-PET abnormalities, the remaining 11 CN+ individuals could be classified as preclinical Alzheimer’s disease stage 1 by the NIA-AA criteria (see Introduction)^9^. We reanalyzed the data restricting it to the 11 CN+ individuals categorized as stage 1, and 30 CN− subjects by eliminating the two outliers of the VSRAD *z*-score, who may possibly represent a preclinical stage of other dementia, such as dementia with grains. We found the performances of the MEG FC markers were almost identical (Supplementary Table S3). These results suggest that MEG can detect subtle functional changes associated with amyloid deposition earlier than metabolic or anatomical changes.

There are several limitations to this study. First, although it is not easy to integrate MEG with amyloid-PET/FDG-PET due to the sparse availability of the devices, the sample size was still small. Larger-scale studies, including subjects with other stages (stages 2 and 3) of preclinical AD, as well as longitudinal observations are necessary to confirm the clinical meaning of the MEG markers. Direct comparisons between the resting state fMRI and MEG are also needed. Second, information for tau markers was not available. The medial temporal atrophy, which is known to be closely associated with the tau pathology^52, 53^, was not observed in CN+ subjects; however, direct tau markers such as CSF or tau PET imaging would be helpful to deepen the interpretation of the MEG results^54^. Third, we focused on only the DMN in this study, because the DMN is one of the key structures for Alzheimer’s disease pathophysiology, and it can reduce the number of possible comparisons that may lead to spurious or complicated results. However, analysis of whole brain dynamics may provide further insights.

In conclusion, this study demonstrated that MEG could be useful as one of the earliest markers downstream to amyloid deposition, providing information distinct from FDG-PET and structural MRI. With the non-invasiveness of MEG, it may also useful in monitoring the effects of interventions in future clinical trials.

## Electronic supplementary material

Supplementary Information
