# Supplementary material for: Early functional network alterations in asymptomatic elders at risk for Alzheimer’s disease
Source: Sci Rep. 2017 Jul 26;7:6517. doi: 10.1038/s41598-017-06876-8 (PMC5529571; doi:10.1038/s41598-017-06876-8)
Supplement: Supplementary file 1 — Supplementary Information [file 41598_2017_6876_MOESM1_ESM.pdf]

Early functional network alterations in asymptomatic elders at risk for Alzheimer's disease

Akinori Nakamura[*+a], Pablo Cuesta[+a,c,d], Takashi Kato[a,b], Yutaka Arahata[b], Kaori Iwata[a], Misako Yamagishi[a], Izumi Kuratsubo[a], Kimiko Kato[a], Masahiko Bundo[a,b], Kersten Diers[f], Alberto Fernández[c,e], Fernando Maestú[c,d], Kengo Ito[a,b]

a. Department of Clinical and Experimental Neuroimaging, Center for Development of Advanced Medicine for Dementia, National Center for Geriatrics and Gerontology, Obu, Japan

b. National Hospital for Geriatric Medicine, National Center for Geriatrics and Gerontology, Obu, Japan

c. Laboratory of Cognitive and Computational Neuroscience, Center for Biomedical Technology, Complutense University of Madrid and Technical University of Madrid, Madrid, Spain

d. Department of Basic Psychology II, Complutense University of Madrid, Madrid, Spain

e. Department of Psychiatry, Faculty of Medicine, Complutense University of Madrid, Madrid, Spain

f. Department of Psychology, Technische Universität Dresden, Dresden, Germany

[+] These two authors contributed equally to this paper

[*] Corresponding Author

Akinori Nakamura, M.D., PhD.

Department of Clinical and Experimental Neuroimaging, Center for Development of Advanced Medicine for Dementia, National Center for Geriatrics and Gerontology, 7-430, Morioka-cho, Obu, Aichi 474-8511, Japan. *E-mail: nakamura@ncgg.go.jp*

Supplementary Materials

Supplementary Methods

Participants' inclusion and exclusion criteria

The major inclusion criteria were: 1) Mini-Mental State Examination (MMSE) score >= 24; 2) Clinical Dementia Rating (CDR) = 0; and 3) a normal Logical Memory II score from the Wechsler Memory Scale–Revised (LM2), after adjustment for individual education levels. Because of the different education systems between the United States and Japan, the individual education levels adjustments were slightly different from those of ADNI2 as follows: a) LM2 (paragraph A, which states the maximum score is 25) >= 9 for 16 or more years of education, b) >= 5 for 10 to 15 years of education, and c) >= 3 for 0 to 9 years of education. Individuals under treatment for any significant medical, neurologic, or psychiatric disease, as well as with any history of a major psychiatric disorder, alcohol dependence, or substance dependence, were excluded. Based on MRI findings, individuals with any clinically significant brain focal legions were also excluded. In addition, we used blood tests to confirm that no participant had abnormal thyroid function or vitamin B1 or B12 deficiency.

PiB-PET: Visual rating and classification

The classification of participants into PiB-positive (CN+) and PiB-negative (CN-) groups was determined by the visual interpretation. This was because we considered that visual interpretation is sensitive enough to detect the very localized amyloid deposition that can occur in the early stage of the amyloid pathology. The method was slightly modified from that reported by Rabinovici et al. [1] as follows: PiB-PET images were visually read by two experienced nuclear medicine physicians (K.I. and T.K.) who were blind to the clinical data. The obtained static images were displayed with a rainbow scale and an inverse gray scale. PiB images were rated as "positive" when the tracer binding in the cortical gray matter was deemed equal to or greater than that in the white matter, and as "negative" when only nonspecific tracer binding in the white matter was observed. If the visual interpretations by the two raters did not match after the independent readings, the cases were discussed and a consensus was reached. In this study, the two raters' judgments were

matched in 43 of 45 (95.6%) cases, and only two cases needed to be discussed. In addition, the consensus decisions for these cases were obtained easily.

PiB-PET: Quantitative image analysis

The reconstructed static PET images (168 x 168 x 111 matrices, 2.036 x 2.036 x 2.036mm voxel size) were spatially normalized in Montreal Neurological Institute (MNI) stereotactic space with parameters obtained from individual 3D-T1 MR images coregistered to PiB-PET images by Diffeomorphic Anatomical Registration Through Exponentiated Lie Algebra (DARTEL) [2]. The normalized PiB-PET images were masked with the grey-matter-segmented MR images to exclude the white matter and regions outside the brain. Then the standardized uptake value ratio (SUVR) images were generated by dividing the masked PiB images by the average value in both cerebellar hemispheres on a pixel-by-pixel basis using the regions of interest (ROIs) of the Harvard-Oxford probabilistic atlas [3]. Mean cortical SUVR (PiB-mcSUVR) was obtained by averaging the SUVRs of the frontal, parietal, and temporal Harvard-Oxford ROIs, except for the primary motor and sensory areas. The PiB-SUVR images were spatially smoothed using a Gaussian kernel filter of 8mm at full width at half maximum. With these smoothed SUVR images, voxelwise regression analysis for MEG FC data with the PiB-SUVR images was performed using SPM8.

FDG-PET: Image acquisition

Prior to the FDG-PET examination, all participants fasted for at least 4 hours. After intravenous administration of ^{18}F-FDG (185 ± 37 MBq), participants were instructed to lie on a bed keeping their eyes opened for a resting period of 30 min in a dimly lit and quiet room. Then a dynamic scan was performed in the 3D mode for 30 min (300 sec frame x 6 times).

FDG-PET: Image interpretation

For image interpretation, the hypo-metabolism in the specific regions was rated as 2+ (definitely present), 1+ (probably present), 0 (equivocal), -1 (probably not present), or -2 (definitely not present). With a rating score greater than 1+, it was deemed to have the

Alzheimer's disease-like regional hypo-metabolism. The raters could also refer to corresponding topographical images of FDG-PET and MRIs. If evaluations of the two raters did not match, the cases were discussed, and a consensus reading was reached. There were only two cases that needed to be discussed.

Structural MRI: Image acquisition

High-resolution 3D T1-weighted images were acquired by the MPRAGE (Magnetization-Prepared Rapid Gradient-Echo Imaging) sequence (TE/TI/TR = 2.51/900/1900 ms, 0.977 x 0.977 x 1.1 mm^3) using a Trio 3T scanner (Siemens), and used for volumetric analysis. Because the T1 images were also used for the MEG source modeling, the landmarks for the MEG coordinate systems nasion and bilateral preauricular points were marked with 5.6 mm-diameter vitamin D capsules (ALFAROL, Chugai Pharmaceutical, Tokyo, Japan) to match the MEG and MRI coordinate systems accurately.

MEG: Measurements

The illuminance of the room was set to about 11 lux. The position of four head-position indicator (HPI) coils attached to the scalp, and each subject's headshape relative to three anatomical locations (nasion and both preauricular points) were defined using a 3D digitizer (Fastrak, Polhemus, VT, USA). Subjects' head movements were monitored by these HPI coils, and eye movements were monitored by the vertical and horizontal EOG with two pairs of bipolar electrodes.

MEG: Data preprocessing

The raw recording data were at first submitted to Maxfilter software (v 2.2, correlation threshold = 0.9, time window = 10 seconds) to remove external noise with the temporal extension of the signal space separation method with movement compensation [4]. The 306-channel system has 102 channel locations, each of which consists of two orthogonal planar gradiometers and one magnetometer. In this study, we used data only measured at 102 magnetometers for the subsequent analysis. Accordingly, all of the magnetometers' resting state signals were automatically scanned for ocular, muscle, and jump artifacts using Fieldtrip software [5], and were visually confirmed by an MEG expert (P.C.). The

artifact-free data were segmented in continuous 4-second fragments (trials). At least 20 clean trials (80 seconds of brain activity) were obtained from all participants and preserved for further analyses. The number of artifact-free trials for CN+ and CN- groups were 40 ± 17 and 47 ± 13, respectively, and there were no significant group differences (Mann-Whitney p = 0.20). To calculate the source reconstruction, the time series was filtered in the following frequency bands: delta (2-3.9 Hz), theta (4.1-7.9 Hz), alpha (8.1-11.9 Hz), beta (12.1-29.9 Hz), and gamma (30.1-55.0 Hz). The filtering was performed with a Finite Impulse Response filter of order 1500 designed with a Hamming window. This filter was applied using a 2-pass procedure over the whole 5-minute registers to avoid phase distortion and edge effects.

MEG: Headmodels and source reconstruction

A regular grid of 2455 nodes, with 1 cm spacing, was created in the template MNI brain. This set of nodes was transformed to each participant's space using a non-linear normalization between the native T1 image (whose coordinate system was previously converted to match the MEG coordinate system) and a standard T1 in MNI space. The forward model was solved with the realistic single-shell model introduced by Nolte [6]. Source reconstruction was performed with a Linearly Constrained Minimum Variance Beamformer [7]. For each subject, the covariance matrix was first averaged over all trials to compute the spatial filter's coefficients, and then these coefficients were applied to individual trials, obtaining a time series per segment and source location.

MEG: Atlas-based analysis of functional connectivity (FC)

In this study, the FC was measured by means of phase-locking value (PLV), in each frequency band. The 6 DMN ROIs included in this study contained 156 nodes. Thus, the starting data set per each subject consisted of matrices with the following dimensions: 156 nodes x 4000 samples x 5 frequency bands x trials. Then, for each frequency band and trial, we calculated the PLV [8] via the following procedure: first, for each node j = 1…156, the phase of the signal $x_j(t)$ was extracted by means of a Hilbert transform: $z_j(t) = x_j(t) + i \cdot Hilbert\left(x_j(t)\right) = A_j(t) \cdot e^{i\varphi_j(t)}$; then, the synchronization between a pair of phases $\varphi_j(t)$ and $\varphi_k(t)$ was calculated with the following expression:

$PLV = \frac{1}{M}\left|\sum_{m=1}^{M} e^{i(\varphi_j(t_m)-\varphi_k(t_m))}\right|$, where M = 4000 is the number of samples in the time series (4 seconds length signals sampled at 1000Hz). Finally, the results were averaged across trials ending up with symmetrical 156 nodes x 156 nodes connectivity matrices per subject and frequency band. To address whether volume conduction could be causing these differences, we calculated the correlation between beamformer weights in both groups to produce an estimate of volume conduction [9]. Beamformer weights did not differ between groups in any frequency band, which makes it unlikely that the functional connectivity differences were caused by volume conduction.

MEG: Statistical analysis for FC

The analytic methodology relied on the cluster based permutation test introduced by Maris and Oostenveld [10] and was carried out independently for each frequency band. The methodology consisted of two steps: (1) an intra-ROI FC analysis that computed the local connectivity within each ROI, and (2) an inter-ROI FC analysis that evaluated the inter-regional connectivity from the PCu ROI, which is known as one of the DMN hubs, to each ROI. In both cases, the procedure was essentially the same. In the intra-ROI analysis, we analyzed the FC of all the nodes contained within a ROI, whereas in the inter-ROI analysis, we focused on the FC between the nodes in the corresponding two ROIs. The procedure started by assessing the FC difference between groups for each pair of nodes using the Mann-Whitney test. The significance of the links was assessed using non-parametric randomization (5000 permutations) testing [11]. Only those links with *p*-values < 0.05 were kept and included in the following steps of the analysis. Then, we aimed to extract a robust significant network, also called a motif in graph theory [12]. These motifs consisted of several consecutive, significant links, which systematically showed a diminished or enhanced FC in the CN+ group compared with the CN- group. We considered a motif to be significant only when 1) at least 25% of the nodes that composed the ROI were involved, 2) at least 10% of the links among them had significant FC differences between groups, and 3) the motifs were connected, that is, a path existed between each pair of nodes in the motif [12]. The first two conditions set the minimum dimensions of the motif, and the third condition fixed a constraint in the morphology,

dismissing the insulated links. If more than two motifs survived within a single ROI or single inter-ROI region, we selected the largest motif as being representative. Then, to control for the multiple comparisons problem, we estimated a proper null distribution of F-values by randomizing the original data. First, we randomized the group's configuration (maintaining the same number of subjects in each group). Next, we shuffled the FC matrices. In each randomized dataset, we extracted the new motifs and then calculated the corresponding F-values. The F-values, computed by ANCOVA adjusted for age, over each motif in the original data set, were compared with the equivalent measure from the randomized data. Therefore, for each motif, the proportion of randomizations with F-values higher than the ones in the original data corresponded to the permutation p-values. Only motifs with $p < 0.05$ were kept as significant motifs.

References

1. Rabinovici, G. D. *et al.* Amyloid vs FDG-PET in the differential diagnosis of AD and FTLD. *Neurology* **77,** 2034–42 (2011).
2. Ashburner, J. A fast diffeomorphic image registration algorithm. *Neuroimage* **38,** 95–113 (2007).
3. Desikan, R. S. *et al.* An automated labeling system for subdividing the human cerebral cortex on MRI scans into gyral based regions of interest. *Neuroimage* **31,** 968–980 (2006).
4. Taulu, S. & Simola, J. Spatiotemporal signal space separation method for rejecting nearby interference in MEG measurements. *Phys. Med. Biol.* **51,** 1759–1768 (2006).
5. Oostenveld, R., Fries, P., Maris, E. & Schoffelen, J.-M. FieldTrip: Open source software for advanced analysis of MEG, EEG, and invasive electrophysiological data. *Comput. Intell. Neurosci.* **2011,** 156869 (2011).
6. Nolte, G. The magnetic lead field theorem in the quasi-static approximation and its use for magnetoencephalography forward calculation in realistic volume conductors. *Phys. Med. Biol.* **48,** 3637–3652 (2003).
7. Van Veen, B. D., van Drongelen, W., Yuchtman, M. & Suzuki, A. Localization of brain electrical activity via linearly constrained minimum variance spatial filtering. *IEEE Trans. Biomed. Eng.* **44,** 867–880 (1997).
8. Mormann, F., Lehnertz, K., David, P. & E. Elger, C. Mean phase coherence as a measure for phase synchronization and its application to the EEG of epilepsy patients. *Phys. D Nonlinear Phenom.* **144,** 358–369 (2000).
9. Brookes, M. J. *et al.* Measuring functional connectivity using MEG: Methodology and comparison with fcMRI. *Neuroimage* **56,** 1082–1104 (2011).
10. Maris, E. & Oostenveld, R. Nonparametric statistical testing of EEG- and MEG-data. *J. Neurosci. Methods* **164,** 177–190 (2007).
11. Ernst, M. D. Permutation Methods: A Basis for Exact Inference. *Stat. Sci.* **19,** 676–685 (2004).
12. Stam, C. J. Modern network science of neurological disorders. *Nat. Rev. Neurosci.* **15,** 683–95 (2014).

Supplementary Figures and Tables

Supplementary Fig. S1

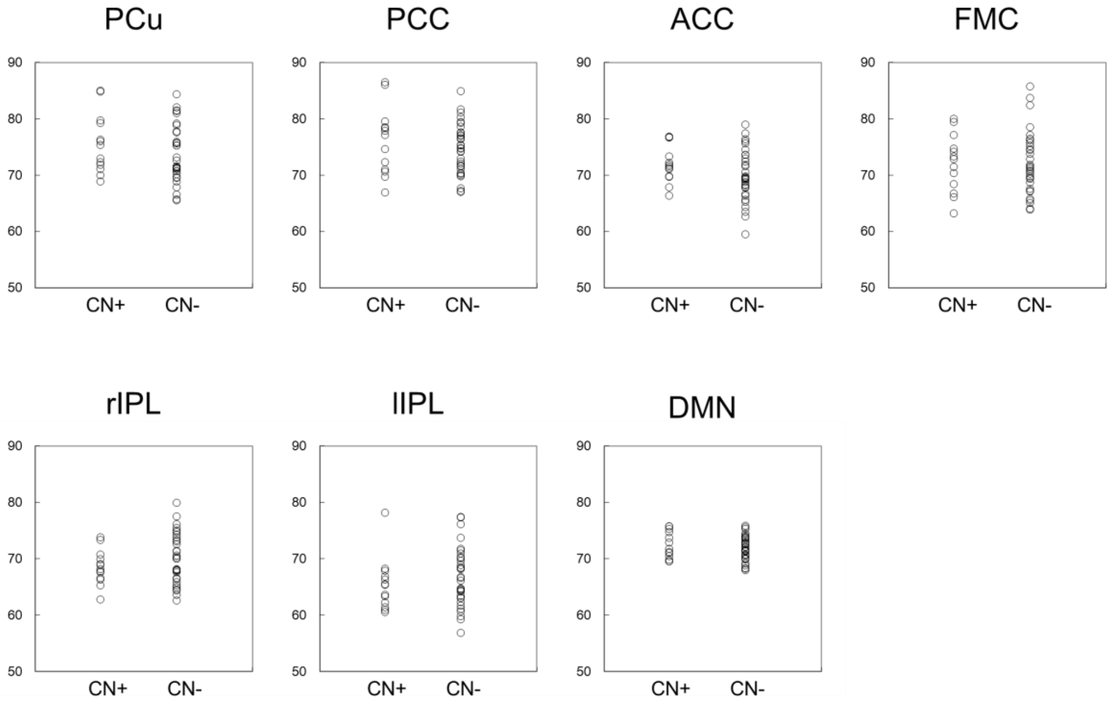

Fig. S1: Scatter plots of the ROI-based volumetric analyses. Y-axes indicate mean value of normalized gray matter volume within each ROI. PCu: precuneus, PCC: posterior cingulate cortex, ACC: anterior cingulate cortex, FMC: frontal medial cortex, rIPL: right inferior parietal lobule, lIPL: left inferior parietal lobule, DMN: default mode network (a merged ROI of these six ROIs)

Supplementary Table S1: Results of the ROI-based volumetric analysis

ROI	CN+	CN-	*t*-test CN+ vs CN- *p* value	Correlation with DMNSUVR (*r*)
Pcu	75.65 ± 5.27	73.76 ± 5.07	0.270	0.107
PCC	76.09 ± 5.98	74.69 ± 4.46	0.393	0.138
ACC	71.95 ± 3.29	69.91 ± 4.53	0.148	0.173
FMC	72.15 ± 5.14	72.12 ± 5.5	0.987	-0.123
rIPL	68.5 ± 3.05	70.05 ± 4.43	0.253	-0.093
lIPL	65.43 ± 4.64	66.75 ± 5.25	0.435	-0.150
ALL_DMN	72.43 ± 2.39	71.91 ± 2.18	0.490	0.042

Values represent mean ± SD. The data correspond to the Fig. S1.

Supplementary Table S2: Effects of the local cortical volume on functional connectivity (FC)

			Unadjusted					Adjusted for cortical volume in the DMN ROI		
			PLV (mean ± SD)		CN+ vs CN- *t*--test	Effect size	Correlation with	CN+ vs CN- ANCOVA	Effect size	Correlation † with
	ROI	Bands	CN+	CN-	*p* value	η_p^2	DMNSUVR (*r*)	*p* value	η_p^2	DMNSUVR (*r*)
Intra ROI FC	PCu	Delta	0.433 ± 0.024	0.470 ± 0.022	< 0.001	0.365	-0.582***	< 0.001	0.370	-0.583***
Inter ROI FC	PCu - rIPL	Delta	0.366 ± 0.008	0.343 ± 0.011	< 0.001	0.519	0.677***	< 0.001	0.519	0.677***
		Theta	0.288 ± 0.015	0.265 ± 0.009	< 0.001	0.500	0.673***	< 0.001	0.524	0.679***
	PCu - lIPL	Delta	0.365 ± 0.011	0.343 ± 0.010	< 0.001	0.504	0.614***	< 0.001	0.507	0.614***
		Theta	0.287 ± 0.009	0.265 ± 0.010	< 0.001	0.533	0.704***	< 0.001	0.570	0.714***
		Alpha	0.264 ± 0.012	0.286 ± 0.014	< 0.001	0.389	-0.473**	< 0.001	0.390	-0.471**

Comparisons of the results of MEG FC markers between unadjusted and adjusted analyses for the cortical volume in the default mode network (DMN) ROIs. Results of the unadjusted analyses are the same as the results shown in Table 2 in the main text, with the exception of the effect size, which was estimated by η_p^2 instead of Cohen's *d*. For group comparison of the adjusted analyses, the ANCOVA was performed using individual cortical volume within DMN ROI as a confounding covariate. Multiple correlation analysis was performed to estimate the linear relationship between the MEG FC values and local Aβ deposition within the DMN (DMNSUVR) adjusted for the cortical volume in the DMN.

† partial correlation adjusted for the cortical volume.

The asterisks indicate statistically significant correlations (** p <0.01, *** p < 0.001).

DMNSUVR: PiB standardized uptake value ratio (SUVR) in the DMN.

Supplementary Fig. S2

CN+ > CN-

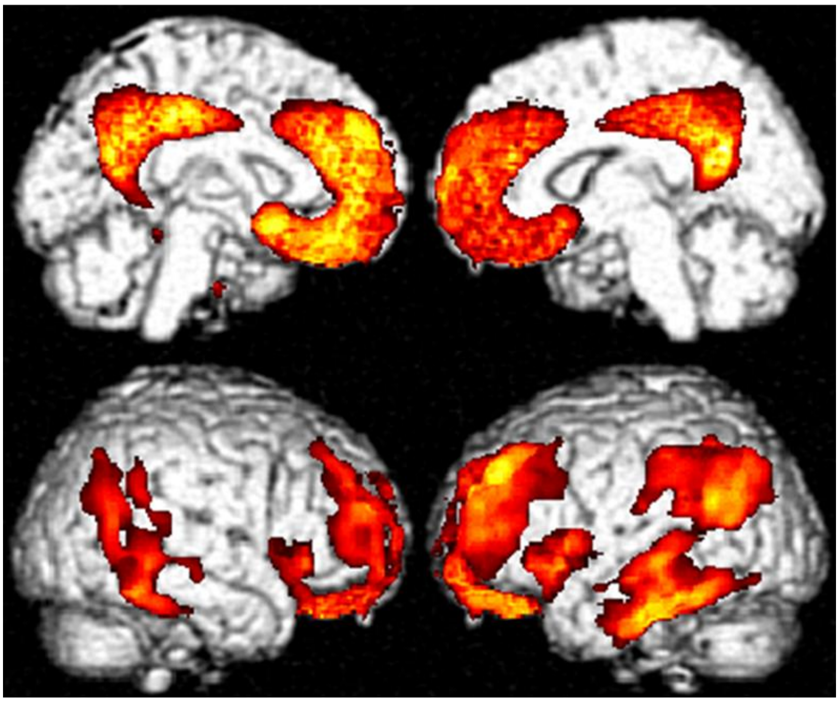

Fig. S2: A group comparison of PiB-SUVR images between CN+ and CN- using SPM8. The height threshold is $p < 0.05$ (FWE corrected), and the extent threshold is $k = 100$ voxels.

Supplementary Table S3: Results of the FC analysis in 11 CN+ and 30 CN- subjects

	ROI	Bands	Number of links (motif size)	PLV (mean ± SD) CN+	CN-	*t*-test *p value* †	Effect size Cohen's *d*	FC changes in CN+ ‡	Correlation with DMNSUVR §
Intra ROI FC	PCu	Delta	39	0.432 ± 0.026	0.47 ± 0.023	< 0.001	1.59	Hypo	-0.581***
Inter ROI FC	PCu - rIPL	Delta	47	0.365 ± 0.008	0.344 ± 0.011	< 0.001	2.10	Hyper	0.620***
		Theta	41	0.287 ± 0.013	0.265 ± 0.009	< 0.001	2.10	Hyper	0.577***
	PCu - lIPL	Delta	29	0.366 ± 0.011	0.342 ± 0.01	< 0.001	2.33	Hyper	0.652***
		Theta	37	0.285 ± 0.008	0.265 ± 0.01	< 0.001	2.15	Hyper	0.697***
		Alpha	39	0.261 ± 0.011	0.286 ± 0.014	< 0.001	1.89	Hypo	-0.564***

†The *p* values were Bonferroni corrected by multiplying with 5 (the number of frequency bands).

‡FC changes in the CN+ group compared with the CN- group. Hypo, decreased connectivity in the CN+; Hyper, increased connectivity in the CN+.

§Correlation coefficient (*r*) for each FC value with mean SUVR value within the DMN ROIs (DMNSUVR). The asterisks indicate statistically significant correlations (* p <0.05, ** p <0.01, *** p < 0.001).

PCu: precuneus, rIPL: right inferior parietal lobule, lIPL: left inferior parietal lobule.
